# How Urban Fringe Expansion Affects Green Habitat Diversity? Analysis from Urban and Local Scale in Hilly City

**DOI:** 10.1155/2022/8566686

**Published:** 2022-09-22

**Authors:** Junyue Yang, Zhong Xing, Canhui Cheng

**Affiliations:** ^1^College of Architecture and Urban Planning of Chongqing University, 400044 Chongqing, China; ^2^College of Architecture and Urban Planning of Guizhou University, 550025 Guiyang, China

## Abstract

Hilly cities in China have gone through an extensive expansion, and urban fringe morphology has experienced a massive change. As a result, green habitats have been occupied or disturbed, and such landscape changes can impact biodiversity. Understanding how urbanization impacts green habitats is essential for urban sustainable development. However, such understanding is lacking for hilly city. This study has two objectives: (1) to quantify the spatiotemporal patterns of green habitats in hilly city fringe during 2000-2020; (2) to identify the differentiated impacts of different hilly city expansion shapes on green habitat. By using landscape indexes to characterize green habitat patterns, the green habitats impact analysis was processed in two scales, at urban scale and local scale. Information Entropy Model and Classification and Green Habitat Quality Evaluation were used to reveal the relationships of urban expansion shapes and green habitat quality in mountainous city. The results showed that, at urban scale, (1) the more complex the city fringe morphology is, the more negative impacts there are on green habitats, (2) and when Guiyang urban fringe green space declined, the green habitats type pattern was refactored. At the local scale, we classified urban fringe expansion into four shape styles; we then discussed the changes of green habitats from the perspective of shape style and stage of urbanization. The results showed that, (1) dispersed type and strip type of urban fringe expansion led to the largest green habitat lost, besides, spreading type and strip type resulted in the largest loss of green habitats core areas. (2) Moreover, at a different stage of urban fringe expansion, the challenge of green habitats persistence was varied, the legacy type has been eager for special species habitats. However, the new type has been facing the risks of guaranteeing habitats stock and quality.

## 1. Introduction

Referring to the environment in which fauna and flora live, a habitat is the sum of ecological factors that affect organisms in a given location. Green habitats refer to green spaces maintaining biodiversity. In urban and rural area, the promotion of biodiversity largely relies on green habitats, the smaller dimensions and fragmented green habitats have particularly significant role to play [1–3]. In particular, the size, heterogeneity, connectivity, and the landscape pattern of green habitat patches have been extensively studied as supporting evidence of urban biodiversity [4–6]. It is worth noting that, urbanization has led to great changes in the urban fringe, results in tremendous changes in green habitats network, then harms urban biodiversity protection [7]. Hence, quantifying the changes of green habitats landscape pattern is crucial for assessment and monitoring of biodiversity consequences of urbanization.

Hilly city refers to cities located in mountainous areas, with cities areas built on more than 15% sloping ground [8]. The landscape layout of green habitats in hilly cities tends to be more fragmented, while the green habitat patches are usually of small scales and high heterogeneity, it is also the reason why mountainous cities enjoy high biodiversity. Such features, being scattered in space and the scarcity of core areas, are also key contributing factors to fragile biodiversity. However, the interactive relationship between urban expansion shape in hilly city and green habitats diversity is still poorly understood.

Land cover dynamics, caused by rapid urbanization, profoundly alter ecosystem services values by occupation or transformation of green habitats [9, 10]. However, the changed patterns of green habitats have obvious gradient characters in urban areas [11, 12]. Due to the high landscape heterogeneity of urban fringe [13], urban fringe is believed to have large landscape multifunctionality, and it is crucial for providing wide range of local ecological services to urban population. Hence, green space sharing strategies should be prioritized in urban fringe areas [12]. Obviously, green habitats change patterns in urban fringe is essential for the understanding of ecological consequences of urbanization.

There are many studies focusing on monitoring and assessing ecological services of green habitats, remote sensing (RS) interpretation and inversion are main land-use and land cover data resources in these studies [14, 15]. Furthermore, landscape analysis is widely applied in spatiotemporal perspective [16, 17], landscape indexes provide an effective approach to present green habitats landscape dynamics, largest patch index (LPI), size of the patch (AREA), and landscape shape index (LSI) are usually adopted to quantify the extent of urban green space extent [18], landscape fragmentation index (LTFI), landscape dynamic index (K), and Shannon-Wiener diversity index (SHDI) are helpful to calculate landscape pattern change and evaluate ecological services of green habitats [19]. In general, green space landscape size, shape, connectivity, quality, and quantity are significant factors for urban ecological security [20].

Besides, equivalent value factor method also contributes to ecological services evaluation of green habitats, the labor theory of equivalent value [21], benefit transfer method [22], equivalent value factors method by crop yield, etc. are commonly used [23–25]. In addition, models are often applied geographically for assessment of variations spatiotemporal effects of urbanization on green habitats fragmentation. Cellular Automata-Markov (CA-Markov) model is useful for green space ecological service value prediction [26], geographically weighted regression model [18] and net primary productivity (NPP) based model [27] are also considered to be effective in assessing variations of urbanization on the fragmentation of green habitats. Mixed analysis is integrated use of methods above, or comprehensive use of land cover data and other types of data, such as nighttime light data (NTL) [18] and land surface temperature data (LST) [28].

On the other hand, remarkably, scaling is critical for habitat analysis. Affected by urban expansion, urban green habitats tend to scatter in distribution and have different shapes, entailing both systematic and level-based scales [29]. It is believed that green habitat pattern analysis at the city and regional level is suitable for habitat assessment and planning, whereas local-scale analysis of the pattern of green space patches should be conducted to verify habitat construction needs. Notably, urban biodiversity is more dependent on the local-scale green habitat system [30], in which the landscape layout features of the green habitats and the intensity of management are both key influencing factors in the distribution of species [31]. Therefore, the observation of changes in the landscape layout of green habitat patches during urban expansion on the local scale is significant to guiding the construction of habitats, managing urban expansion, and integrating urban habitat layout on various scales [32]. Such understanding of habitat changes on the local scale also helps to understand the variation of spatial patterns in cities, which in turn, is important for urban-scale habitat evaluation that serves as a reference for eco-enhancement in cities [33].

China launched a strategy entitled the Delineation and Defense of Ecological Protection Red Lines (EPRLs) to respond to drastic urban and rural construction and inefficient land use [34]. The strategy identified the scope of construction prohibited areas and other protection regions, and assessed the ecological functions, vulnerability or sensitivity of area, EPRLs are the minimum areas that maintains national ecological security [35]. To explain in more detail, national government drafted completed management policies including permitted constructions, ecological compensation, and monitoring and regulation. Provincial government is responsible for delineation of EPRLs, and then executes the scheme of EPRLs [36]. Clearly, EPRLs is the response of maintaining ecological services at national and regional scale, due to the small scale and fragmented green habitats in urban fringe are not in the scope of EPRLs, it is difficult to conduct this strategy at local level.

Most of the previous green habitat studies focused on the relationship between urbanization and pattern of green habitats in plain city [19, 37]. However, hilly city has unique pattern of urban expansion [8], and the relationship between urbanization and green habitats changes in mountainous city is still poorly understood.

This paper studies the changes in the landscape layout of green habitats during urban expansion. The research scope is the urban fringe of hilly cities, where the demand for land is causing increasing tension [38, 39]. Scales are being introduced as key factor to habitat analysis with regard to the factors in green habitats. The interdependence of “urban-local” scales is highlighted. The results of habitat evaluation under the urban scale are adopted as guidance to find out the triggering factors to urban sprawl patterns and the changing habitat details in patch level under the local scale.

The study adopts spatial statistics method [40]. Remote sensing+GIS platform are used to get data on land cover [41]. The landscape entropy model is applied to denominate urban fringe areas. The data are analyzed using FRAGSTATS software and GIS space calculation to evaluate habitat quality on the urban scale, and analyzed the changes in habitat landscape layouts on the local scale [42]. Multiscale spatiotemporal analysis of the landscape layouts in the green habitats is hence conducted to facilitate the comparison of various types of urban sprawl patterns. The feature of changes in green habitat layouts during urban fringe expansion in hilly cities is summarized, after that is the discussion of the impacts of different urban sprawl patterns had on green habitats, and their characteristics in various stages of an urban extension. Recommendations for habitat protection and urban development in hilly cities are also made.

## 2. Region Features and Data Preparation

### 2.1. Region Features

Guiyang is a typical hilly (mountainous) city, over 59% of the city covered with slope over 15°, which meets the definition of mountainous area from the United Nations Environment Programme–World Conservation Monitoring Center [8]. Guiyang's urbanization is different from those of topographically flat urban regions, urbanization in mountainous area has greater dominance of leapfrog expansion mode with smaller and more regularly shaped patches [43]. Guiyang is known as “Lin Cheng”, or the city of forests, it is also the first winner in China of the title “national forest city” and enjoys rich biodiversity. From 2000 to 2020, built-up land in Guiyang increased from 163.97 square kilometres to 467.92 square kilometres, an increase of 285%; in the meantime, the area of agricultural land, woodland, shrubland, and grassland was decreased by 187.68 square kilometres, 58.46 square kilometres, 10.07 square kilometres, and 61.00 square kilometres, respectively. Those numbers have shown that the city has expanded significantly in the past two decades, resulting in a massive encroachment on greenfield habitats [44]. Typical urbanization impacts, such as the fragmentation of habitat and quality degradation, are seriously threatening urban biodiversity in Guiyang and eco-security in the region [45, 46].

### 2.2. Data Preparation

**Figure 1:**
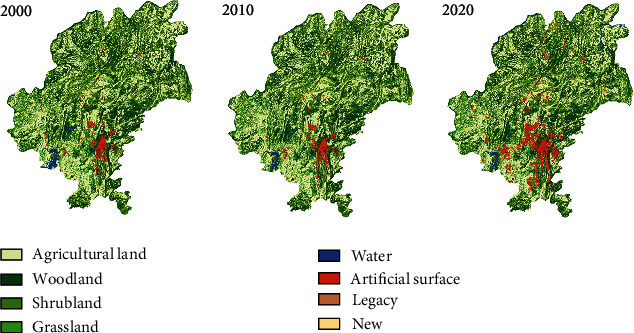
Land Cover in Guiyang in 2000, 2010, and 2020 (data source: Globeland30).

Data used in this paper is the Landsat ™ 30 m × 30 m remote sensing data from the Globeland30 (Global Geographic Information Public Product) platform, which has been decoded and calibrated for land cover (see Figure 1). The data is imported into ArcGIS10.2 to create a local sample area of 2 km × 2 km of the Guiyang city, which includes 2,674 grids. The Guiyang land cover data for 2000, 2010, and 2020 are cut by and aligned with the grids. In total, there are 8,022 entries in the grid property sheet, whose landscape layout indexes are calculated using FRAGSTATS 4.2 simultaneously.

## 3. Research Methods

### 3.1. Scoping Urban Sprawl

#### 3.1.1. Landscape Entropy Model

The landscape in urban fringe areas is highly heterogeneous due to diverse, fragmented, and highly variable land use. Landscape entropy is a quantifiable tool to evaluate the degree of landscape heterogeneity and disturbance. From an urban-rural comparison perspective, the artificial landscape patches in the urban centre and the natural landscape patches in the urban periphery are more homogeneous in type, resulting in low landscape disturbance [47]. Urban fringe, nonetheless, is defined as a horizontally embedded area of the city, which is viewed as a patch of artificial disturbance, on the ecological background [48]. Featuring a mixture of land use, building types, and fragmentation of landscape patches, urban fringe areas usually show high disturbance. This study uses an information entropy model to define the urban fringe areas in Guiyang. The equation is as follows:

(1) $$\begin{array}{c} W=-\overset{n}{\underset{i=1}{\sum }}X_{i}\ln\;X_{i}. \end{array}$$

In the equation, *W* is the entropy value of landscape disturbance, the larger *W* is, the more disordered the landscape; *X*_*i*_ is the percentage of area occupied by a certain type of land use, and *i* = 1, 2, 3⋯, which equals to the number of land use types. The calculated entropy values for landscape disturbance in Guiyang were 0-0.73 in 2000, 0-0.82 in 2010, and 0-0.75 in 2020.

#### 3.1.2. Determining Urban Inner and Outer Edges

**Figure 2:**
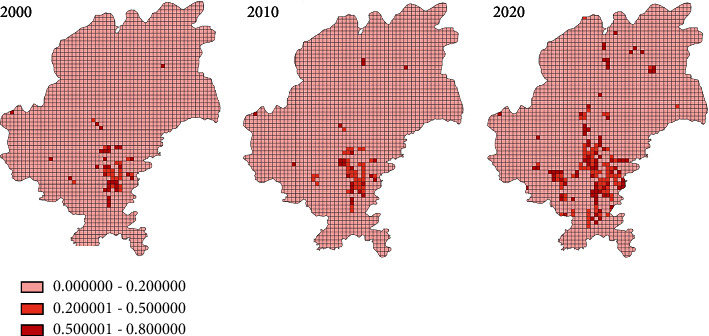
Urban fringe areas of Guiyang in 2000, 2010, and 2020.

After analyzing the spatial distribution in urbanized Guiyang and the percentage of artificial surface, we picked the following criteria for inner and outer boundaries of the urban area: entropy value less than 0.2 while the percentage of artificial surface greater than 50% (inner, to exclude urban area); and the percentage of artificial surface greater than 25% (outer, to exclude the influence of the high entropy value of landscape disturbance caused by fragmented mountainous farmlands and other landscapes). Thus, the time-space variation of the urban fringe area in Guiyang is shown (as in Figure 2).

### 3.2. Influence Analysis of Habitats in Urban Fringe Areas (Urban Scale)

#### 3.2.1. Selecting Habitat Quality Evaluation Factors

The size of habitat patches is the key to biodiversity, as the patch sizes gets larger, they become more capable of maintaining biodiversity. Habitat diversity can therefore be evaluated for continuation by quantifying the size changes of different types of habitats and the size distribution of habitat patches. Besides, given that large habitat patches are rare in urban fringe areas of hilly cities, whereas some species are sensitive to the size of their habitats and only live in the core area of habitat patches [49, 50], calculating the changes in the sizes of core areas in habitat patches can serve as an indirect evaluation of the survival of those species. Moreover, we believe that considering the difficulties caused to animal migration by fragmented and scattered landscape patterns of mountainous green habitats, the accessibility among habitat patches of the same type becomes crucial to securing biodiversity [51]. Consequently, the following landscape indicators at the patch level are selected as key indicators to evaluate green habitat quality, based on the features of green habitats in hilly cities and the continuation dilemma they face: AREA (size of the patch), CORE (size of the core area), and PROX (the proximity of patches). Among those key indicators, PROX represents the closeness in geography of a certain patch to another of the same type at the patch level. To enable its calculation, the search distance is set at 500 meters with the reference patch as the center in advance. The calculation equation of PROX is shown as follows:

(2) $$\begin{array}{c} PROX=\overset{n}{\underset{s=1}{\sum }}\frac{a_{ijs}}{h_{ijs}^{2}}. \end{array}$$

In the equation, a_ijs_ is the size of the adjacent area between patch i_js_ and patch i_j_, and the h_ijs_ is the distance between patch i_js_ and patch i_j_, and the distance between patch edges is calculated as the distance between the meta cell and the meta cell center. The larger the PROX value, the closer the given patch is to other patches of the same type.

#### 3.2.2. Assignment Weight Using CRITIC

The CRITIC method, which gives priority to the comparative strength of indicators and the conflicts between indicators, is a good choice for assigning weight to indicators in an attempt to evaluate the quality of habitats. For example, if the total patch size contrasts strongly against the core area size in a certain type of habitats, it means that that type maintains better diversity, which is translated into higher weighting. If the indicators are more positively correlated, it means that they contrast less, and the information reflected in the evaluation shows more resemblance, which will reduce its weighting. The standard variations of the indicators are calculated to express their variability, while the correlation coefficient is calculated to find out the degree of contrast among those indicators. The amount of information is determined as the product of the representation of variability and contrast degree, while the final weighting is decided by applying normalization calculation to the amount of information. The results are shown in Table 1.

#### 3.2.3. Habitat Quality Evaluation

In the years concerned in this study, i.e., 2000, 2010, and 2020, the majority of green habitat in urban fringe areas in Guiyang was used as agricultural land, woodland, shrubland, and grassland. The following equation is applied to calculate the habitat quality of each of the patches at the patch scale:

(3) $$\begin{array}{c} E_{i}=A_{i}w_{A}+C_{i}w_{C}+P_{i}w_{P}. \end{array}$$

**Figure 3:**
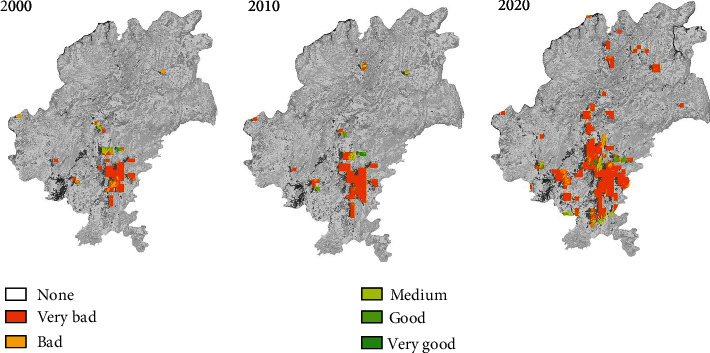
Habitat quality evaluation, urban fringe area, Guiyang, in 2000, 2010, and 2020.

In the equation, *E*_i_ is the habitat quality indicator of patch *i*; *A*_i_ is the size of patch *i*, and *w*_A_ is the index weight given to patch size; *C*_i_ is the size of the core area of patch *i*, and *w*_C_ is the index weight given to the core area size; *P*_i_ is the proximity index of patch *i*, and *w*_P_ is the index weight given to the proximity index. The results of the quality evaluation are divided into five levels: very good, good, medium, bad, and very bad. GIS is used to visualize the results and generate the diagrams that represent the habitat quality in the urban fringe area of Guiyang in the three years concerned, respectively. (As shown in Figure 3).

#### 3.2.4. Morphological Change Analysis of Urban Fringe

The landscape indicator ED (edge density) is introduced to describe the complexity of edge shapes. ED is calculated by the total edge length divided by the total area size. The higher the value, the more complex the edge shape is. It is shown in Table 2 that ED values registered an upward and then downward trend. In the first decade when urban expansion was slow, the shape of the edges became complicated gradually, whereas in the second when it was rapid, the complexity of the edges was lowered.

### 3.3. Change Analysis of 2 km ×2 km Green Habitat Grids (Local Scale)

#### 3.3.1. Identifying Samples and Classification

Typical sample patches with radical changes in habitat quality and land use are screened out under the local scale. Some sample patches identified in the 2000 data set remained in that of 2010 and 2020. They experienced slow expansion while keeping the space features of urban fringe areas, i.e., high entropy value of landscape disturbance, which are defined as the legacy type. On the other side, from 2010 to 2020, the urban fringe of Guiyang expanded rapidly, causing the sprawl to reshape accordingly. The samples that produced at this stage are defined as the new type. Therefore, the typical sample patches are sorted into two types according to their time features, the legacy type and the new type, and they are divided into four types, dispersing, strip, spreading, and enclosed, according to their shape features after expansion. See Table 3 for details.

#### 3.3.2. Understanding Spaciotemporal Changes in Green Habitat in Typical Sample Patches

**Figure 4:**
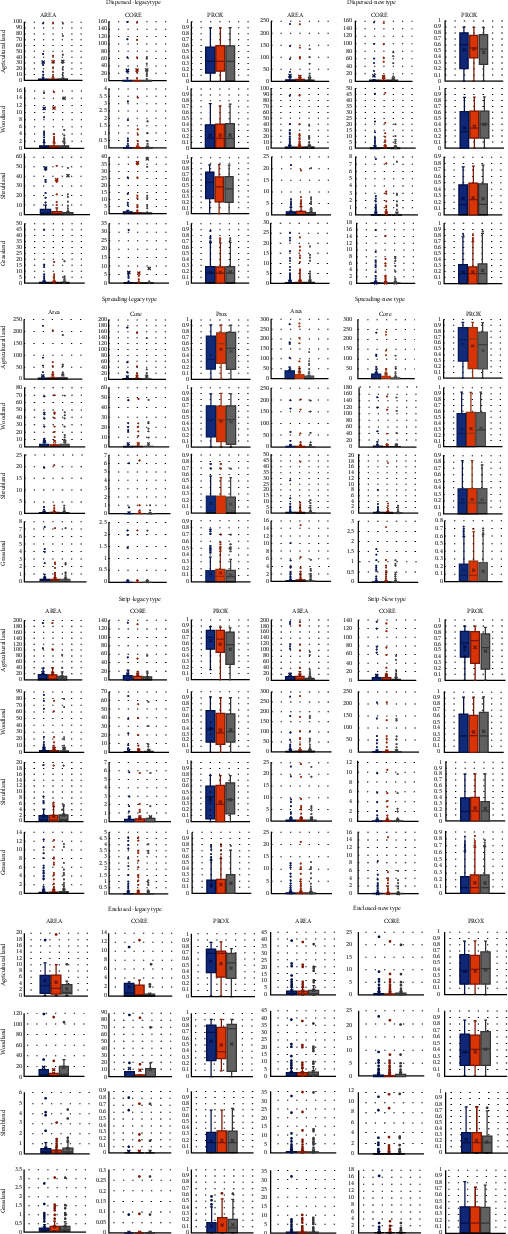
Habitat quality analysis of typical sample patches.

The landscape indicators are aggregated categorically and expressed in box plots, which help to show the distribution of patch size, core area size, and proximity values of different types of sample areas. Data from different years were integrated and analyzed for a comparative study of habitat changes caused by the various types of urban expansion to shed light on size changes, the continuation of core areas, and proximity changes of same-type habitat patches under different types of sprawl and urban expansion stages. (See Figure 4).

## 4. Results and Analysis

### 4.1. Urban Scale Analysis on Green Habitat Changes

#### 4.1.1. Relevance between Edge Shapes and Green Habitat Quality Changes

From 2000 to 2010, the edge shapes in the city got complicated and habitat quality deteriorated significantly. However, from 2010 to 2020, the newly added urban sprawl edges showed decreased complexity and the corresponding urban fringe areas hosted more patches of good and medium quality. It is therefore concluded that the changes in urban edge shapes are related to the changes in habitat quality. The more complicated the urban edge shapes, the worse the habitat quality in the corresponding areas. (See Table 4).

#### 4.1.2. Analysis of Habitat Layout Changes

Observing from the urban scale, green land habitats experienced quality deterioration during the 20 years featuring reduced size, shrinking core area size, and reduced patch proximity, which resulted in a remarked change in the area and type distribution of habitats.

**Figure 5:**
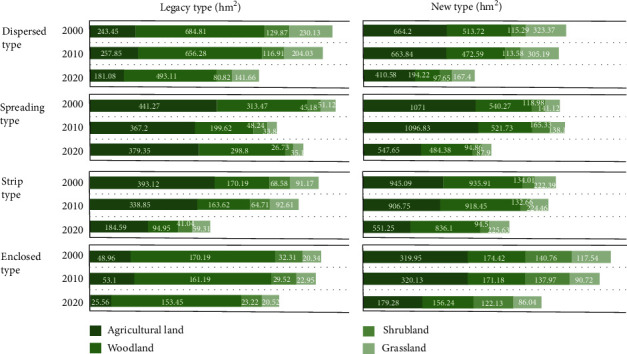
Statistics of classified size results of typical samples.

From the size distribution of habitats perspective, most habitats in the urban fringe of Guiyang are small-sized habitat patches (see Figure 5). Over the 20 years, the concentrated distribution range (more than 99.3% of total data) of agricultural land patches decreased from 0-58 hm^2^ to 0-30 hm^2^; woodland patches, 0-15.5 hm^2^ to 0-8.5 hm^2^; shrubland patches, 0-4.2 hm^2^ to 0-3 hm^2^; and grassland patches, 0-1.5 hm^2^ to 0-1.2 hm^2^. It is concluded that small patches of all types were becoming even smaller, and some of the tiny patches disappeared. It has always been clear that the “reverse T shape”, with the dominant patches being small and large patches being rare, was kept.

Agricultural and woodland are major types of habitats in the urban fringe of Guiyang, which dominate the biodiversity changes. As it is shown in Figure 5, the four space shape expansion types show similar results, i.e., agricultural land/woodland> woodland/agricultural land>grassland/shrubland>shrubland/grassland. It is worth pointing out that due to continued urbanization, the core areas of shrubland and grassland have been almost eliminated in the urban fringe of Guiyang, meaning that species sensitive to those habitats are facing extinction risks in the urban sprawl. As shown in Figure 4, the concentrated distribution analysis discovered that the core area size of both shrubland and grassland habitats are both zero, with the only exception in outlier distribution where a few of core areas are kept. However, they still are being reduced in the process of urban expansion.

### 4.2. Local Scale Analysis on Habitat Changes

#### 4.2.1. Differentiated Impacts of City Expansion Shapes on Habitat

In the case of Guiyang, the dispersing and strip shapes of urban fringe expansion made most encroachment of habitats (see Table 5): dispersing (1138.32 hm^2^) > strip(873.09 hm^2^) > spreading(767.61 hm^2^) > enclosed(258.03 hm^2^). At the same time, the spreading and strip shapes of urban fringe expansion made most encroachment of core habitat areas: dispersing (610.74 hm^2^) > strip (540.89 hm^2^) > spreading(540.83 hm^2^) > enclosed(246.13 hm^2^). However, the change in urban fringe expansion shapes did not have a significant differential impact on the proximity of habitat patches.

#### 4.2.2. Impacts on Habitats of Urban Expansion Stages

Habitats in legacy and new urban fringe areas are exposed to distinct continuation risks. The legacy type largely consumes small-size green habitat in its slow expansion, whereas the rapidly expanding new type encroaches heavily on large areas of green habitat. Such comparison is highlighted in the changes we found in woodland and shrubland habitats. As shown by Table 6, among sample patches in legacy areas, the woodland habitats show the highest percentage of core area size vis-à-vis the total area (63%), and the maximum value of concentrated distribution of core area size increased by 12.20 hm^2^, meaning that the number of small woodland patches with core areas sharply decreased in legacy areas. In contrast to that, woodland patches in new areas lost (46%) far less core areas than in the legacy areas, and the max value of core area concentrated distribution dropped by only 0.60 hm^2^, a signal that large-size woodland patches in new areas are more seriously encroached than their peers in the legacy areas. The shrubland habitats, however, lost 30% of core areas in new areas and a mere 16% in legacy areas.

We gather that the perpetual loss of small-sized core areas is exposing legacy type urban fringe areas to even more serious species continuity crisis in terms of biodiversity in the sense that species that are particularly sensitive to habitat size are disappearing in the legacy type of urban fringe areas. In the meantime, living spaces of wildlife in the new type areas are undergoing extensive compression, making it a priority to securing sufficient and effective spaces to guarantee biodiversity. In other words, in the legacy areas, it is urgent to solve the problems of “having or having not” habitats for sensitive species, while the new type areas should give more attention to the “enough or not” and “good enough or not” issues.

## 5. Discussions and Conclusion

### 5.1. Discussion

The results of this study show that, on urban scale, (1) the more complex the expansion form of hilly city fringe, the more obvious the negative impacts on green habitats, (2) green habitat type patters in Guiyang changed obviously as the size of green habitats sharply decreased in Guiyang urban fringe. On local scale, (1) dispersing type and strip type of urban fringe expansion caused the most serious habitats areas lost; spreading type and strip type resulted in largest loss of habitats core areas lost, (2) the habitat continuity risks facing the legacy and new type of urban fringe areas are different: the legacy type should urgently solve the problems of “having or having not” habitats for sensitive species, while the new type areas should prioritize the “enough or not” and “good enough or not” issues. Hence, the study advocates three strategies for hilly city fringe shapes control and green habitat building.

#### 5.1.1. First Aid to Endangered Habitats and Habitat Pattern Conservation Guided by Dominant Habitats

As suggested by the results of this study, diverse types of habitats face distinctive dilemmas in habitat retention during urban expansion, and curating efforts should give more attention to the protection and restoration of habitat patterns according to types while promoting an overall network of green spaces.

As agricultural and wood land areas consistently account for the largest proportion in the urban fringe area of Guiyang during urban expansion, they dominate the urban biodiversity patterns of the area. On the contrary, shrublands and grasslands account for a small proportion; their patches scattered and tiny. Not only so, but their core areas are also on the verge of exhaustion and demonstrate a low proximity index, rendering risks of extinction for wildlife in these two types of habitats in the urban fringe. In terms of biodiversity conservation in urban fringe of Guiyang, first aid to endangered habitats should be a top priority for habitat pattern conservation.

Apart from that, we believe that as dominant habitat forms, agricultural and woodland areas are positioned to become the skeleton of the habitat layout, which, if built jointly with other habitats and organically combined with the industrial, livelihood, and eco-protection areas, will become an effective foothold to habitat layout protection in hilly cities as the fragmented small patches of habitats can be connected to empower a habitat system.

#### 5.1.2. Urban Fringe Pattern Optimization and Control under Multiple Solution Comparison

The habitat depletion patterns have a key impact on the continuation of species ^[16]^. In other words, the choice of urban expansion patterns is critical to the protection of biodiversity. Multiscale habitat change analysis based on landscape layout indexes are making the multiscenario simulation of urban expansion pattern a reality. Considering that additive planning, replanning of existing urban areas, and planning adjustments are indispensable for hilly cities, it is a key step to protect biodiversity that urban space patterns are put under control.

#### 5.1.3. Differentiated Setting of Green Habitat Building Objectives from an Area-Based Perspective

Urban fringe areas face various biodiversity risks in respective urbanization stages. For instance, in the legacy type of urban fringe areas of Guiyang, the continuity of species is threatened, while in the new type areas the risk of the decimation of biological populations is getting more severe. Consequently, the targets set in each type of urban fringe area should be different accordingly. In legacy type areas in Guiyang, the core target should be the protection of habitat diversity, to which the new type areas should add all types of bottom-line habitat size targets.

### 5.2. Conclusion

In order to explain the spatiotemporal changes of green habitats in urban fringe under the unique urban expansion pattern of hilly city. The study selected landscape indexes of AREA (size of the patch), CORE (size of the core area), and PROX (the proximity of patches) to present the quality of green habitats in hilly city fringe. The calculation was conducted at urban scale to reveal the urban expansion pattern and green habitat type patters changes during 2000-2020. For clarifying the relationship between urban expansion pattern and green habitats changes, the detailed calculation was conducted at local level basing on classification of urban fringe expansion shapes. Then strategies on urban fringe shapes control and green habitat building in hilly city were given. It is safe to say that, urban and local scale integrated analysis of green habitats is necessary for understanding the interaction of green habitats and urbanization in hilly city, and the local scale analysis made a great deal of contributions on finding key problems of green habitats maintaining.

The method combining the use of GIS analysis, FRAGSTATS software, landscape entropy model and landscape indexes offered a new way of thinking and methodology for the analyzing the changes of green habitats caused by urbanization in hilly city fringe. Meanwhile, this research also promoted the quantitative analysis of green habitats research, and enriched the hilly city case in urban green habitat research.

The results confirmed that the unique urban expansion pattern of hilly city led to special green habitat evolution consequences, both the quality and structure of the green habitat were affected, such changes were closely related to the shapes of urban fringe and the stage of urbanization. This conclusion has strong potential on leading urban space management policies and the coherence of city fringe green habitats and urban sustainable development.

## Figures and Tables

**Table 1:** Results of CRITIC Method.

Item	Variability	Deg. of contrast	Amt. Of info.	Weight
AREA	13.517	0.958	12.956	18.63%
CORE	7.958	0.959	7.633	10.98%
PROX	25.921	1.889	48.954	70.39%

**Table 2:** Edge Changes in Urban Fringe.

Year	Area (hm^2^)	Total edge(m)	ED(m/hm^2^)
2000	22444.333	1966388	87.612
2010	26421.813	2344377	88.728
2020	67776.610	5603412	82.674

**Table 3:** Grouping of typical sample patches.

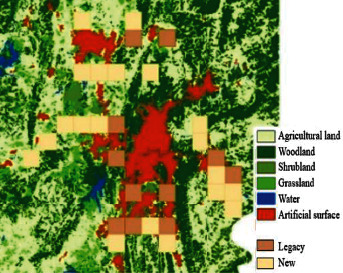	Type	Legacy	New
	Spreading	Sample 1, 2, 18	Sample 22, 24, 25, 27, 30, 39
	Strip	Sample 6, 12, 14	Sample 26, 28, 31, 32, 36, 40
	Enclosed	Sample 7	Sample 23, 33, 38
	Dispersed	Sample 9, 11, 13, 16, 17	Sample 21, 29, 34, 35, 37

**Table 4:** Changes in urban fringe areas.

Year	Very bad to medium quality (%)	Very good, good quality (%)	ED
2000	50.81	49.19	87.612
2010	52.62	47.38	88.728
2020	49.52	50.48	82.674

**Table 5:** Reduction of habitat size and core area (in types).

	Legacy type				New type			
	Dispersed type	Spreading type	Strip type	Enclosed type	Dispersed type	Spreading type	Strip type	Enclosed type
Decrease of AREA (hm^2^)								
Agricultural land	62.37	61.92	208.53	23.40	253.62	523.35	393.84	140.67
Woodland	191.70	14.67	75.24	16.74	319.50	55.89	99.81	18.18
Shrubland	49.05	18.45	27.54	9.09	17.64	24.12	39.51	18.63
Grassland	88.47	16.02	31.86	-0.18	155.97	53.19	-3.24	31.50
In total	391.59	111.06	343.17	49.05	746.73	656.55	529.92	208.98

Decrease of CORE AREA (hm^2^)								
Agricultural land	33.65	54.72	107.55	14.76	169.20	500.85	294.12	157.68
Woodland	129.51	8.10	38.43	12.36	121.06	23.49	49.95	31.54
Shrubland	5.58	5.22	4.95	0.63	2.02	8.91	10.91	8.28
Grassland	41.08	4.42	12.78	-0.18	38.79	6.03	22.14	21.06
In total	209.82	71.46	163.71	27.57	331.07	539.28	377.12	218.56

Decrease of PROX (%)								
Agricultural land	51.10	42.30	68.70	28.80	13.00	78.40	89.50	70.30
Woodland	-109.00	-9.10	0.00	59.40	76.05	-4.80	12.90	1.81
Shrubland	40.05	7.05	56.20	42.30	-6.10	-10.05	14.30	10.29
Grassland	6.05	26.30	16.60	-89.40	56.50	11.70	-16.70	37.50

**Table 6:** Landscape indicator changes of legacy type and new type.

	Legacy type				New type			
	Agricultural land	Woodland	Shrubland	Grassland	Agricultural land	Woodland	Shrubland	Grassland
Decrease of AREA (hm^2^)	356.22	298.35	104.13	136.17	1311.48	493.38	99.90	237.42
Decrease of CORE AREA (hm^2^)	210.63	188.04	16.38	57.10	1121.85	226.04	32.12	88.02
The ratio of reduced core area to total area	0.59	0.63	0.16	0.42	0.86	0.46	0.30	0.37
Decrease of MAX PROX	1.91	-0.58	1.46	-0.40	2.51	0.86	0.09	0.89
Decrease of MAX concentrated distribution AREA	23.60	-19.50	1.48	-0.45	60.00	-1.10	0.70	0.30
Decrease of MAX concentrated distribution CORE AREA	12.65	-12.20	0.00	0.00	52.50	0.60	0.00	0.00
Decrease of MAX AREA outlier	165.00	142.00	27.50	27.40	450	152.00	16.00	36.00
Decrease of MAX CORE AREA outlier	114.90	82.00	6.50	30.32	380	84.00	4.20	25.50

## Data Availability

All data used in this study are presented in the manuscript.
